# Predictors of futile recanalization in patients with acute ischemic stroke undergoing mechanical thrombectomy in late time windows

**DOI:** 10.3389/fneur.2022.958236

**Published:** 2022-09-15

**Authors:** Heng Ni, Xinglong Liu, Yu Hang, Zhenyu Jia, Yuezhou Cao, Haibin Shi, Sheng Liu, Linbo Zhao

**Affiliations:** Department of Interventional Radiology, The First Affiliated Hospital of Nanjing Medical University, Nanjing, China

**Keywords:** futile recanalization, mechanical thrombectomy, acute ischemic stroke, late time windows, predictors

## Abstract

**Background and purpose:**

Futile recanalization (FR), defined as functional dependence despite successful reperfusion, is common in patients who experience an acute stroke after thrombectomy. We aimed to determine the predictors of FR in patients who underwent thrombectomy in late time windows (6 h or more after symptom onset).

**Methods:**

This retrospective review included patients who underwent thrombectomy for acute anterior circulation large vessel occlusion from October 2019 to June 2021. Successful reperfusion was defined as a modified Thrombolysis in Cerebral Infarction (mTICI) score of 2b/3. Functional dependence at 90 days was defined as a modified Rankin scale score of 3–6. Multivariate analysis and a receiver operating characteristic (ROC) curve were used to identify the predictors of FR in patients treated in delayed time windows.

**Results:**

Of the 99 patients included, FR was observed in 51 (51.5%). In the multivariate analysis, older age (OR, 1.12; 95% CI, 1.04–1.22; *P* = 0.005), female sex (OR, 3.79; 95% CI, 1.08–13.40; *P* = 0.038), a higher National Institutes of Health Stroke Score (NIHSS) score upon admission (OR, 1.11; 95% CI, 1.02–1.22; *P* = 0.023), and an increased number of passes per procedure (OR, 2.07; 95% CI, 1.11–3.86; *P* = 0.023) were independently associated with FR after thrombectomy. The ROC curve indicated that the model that combined age, female sex, baseline NIHSS score, and the number of passes per procedure (area under the curve, 0.84; 95% CI, 0.75–0.90, *P* < 0.001) was able to predict FR accurately.

**Conclusions:**

Older age, female sex, higher NIHSS score upon admission, and an increased number of passes per procedure were independent predictors of FR in patients who experienced acute ischemic strokes after thrombectomy in late time windows.

## Introduction

Mechanical thrombectomy (MT) is widely accepted as a standard approach for acute large-vessel occlusions (LVOs) in the anterior circulation in patients with acute ischemic stroke (AIS) (1). Recently, the results from the DAWN and the DEFUSE-3 trials have demonstrated the safety and efficacy of MT in late time windows in patients with AIS selected by perfusion imaging (2, 3). However, a substantial proportion of patients experience futile recanalization (FR; defined as poor clinical outcomes despite successful recanalization) after thrombectomy in late time windows, even though these patients are screened with rigorous imaging (2–4). A real-world study showed that patients who underwent endovascular treatment more than 6 h after symptom onset had a relatively higher rate of poor outcomes despite successful reperfusion compared with those treated within 6 h of symptom onset (4). Thus, predicting FR in these specific populations could help select a population of patients treated with MT that would potentially benefit more of adjunctive therapies to maximize the benefit of MT.

Many previous studies have identified predictors of FR after endovascular treatment in early time windows in patients with AIS (5–12). However, few studies have investigated the predictors of FR in patients treated with MT in late time windows. Therefore, this study aimed to identify the potential predictors of FR in patients with AIS who underwent thrombectomy in late time windows.

## Materials and methods

### Population selection

**Figure 1:**
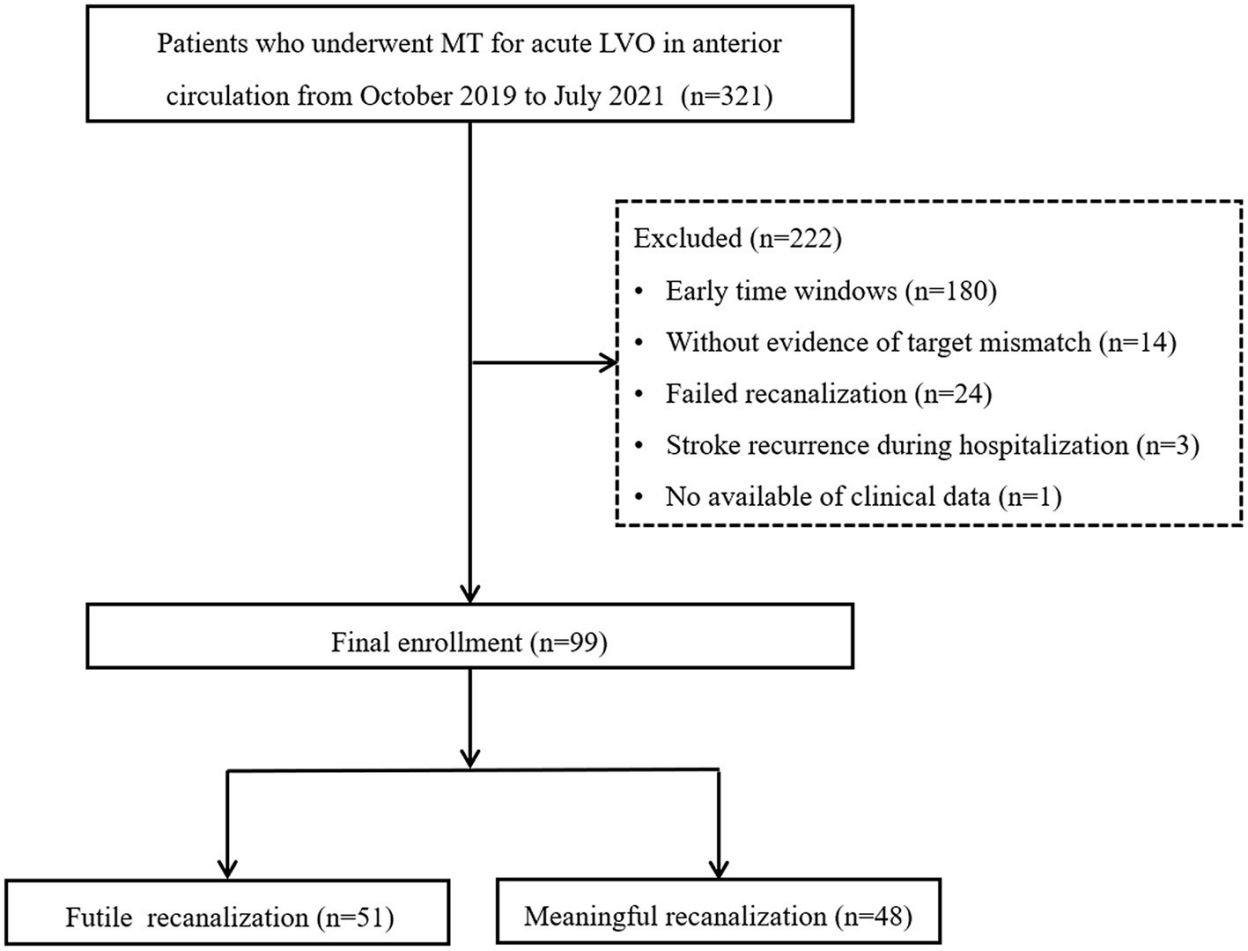
Flowchart of patient selection. MT, mechanical thrombectomy; LVO, large vessel occlusion.

From October 2019 to June 2021, 321 patients who underwent MT for acute LVO in the anterior circulation were retrospectively reviewed using the stroke database. The selection criteria were as follows: (1) age of ≥18 years; (2) a modified Rankin scale (mRS) score of 0–1 before stroke; (3) an initial National Institutes of Health Stroke Scale (NIHSS) score of ≥6; (4) MT performed 6 to 24 h after symptom onset; (5) occlusion of the internal carotid artery (ICA) and/or middle cerebral artery (MCA) M1 or proximal M2; (6) fulfillment of DAWN or DEFUSE-3 criteria in CT perfusion (CTP) imaging; and (7) successful recanalization. The exclusion criteria were as follows: (1) occlusion of posterior circulation or anterior cerebral artery; (2) MT performed within 6 h of symptom onset; (3) failed recanalization; (4) stroke recurrence during hospitalization; and (5) loss of clinical data or follow-up results. Ultimately, 99 patients were included in the study. This study was approved by the local institutional review board, and because of the retrospective study design, the requirement for informed consent from patients was waived. Figure 1 shows the workflow for patient selection.

### Clinical and imaging data evaluation

The following clinical data were collected: demographic features (age and sex), medical history (hypertension, hyperlipidemia, diabetes mellitus, myocardial infarction, atrial fibrillation, smoking history, ischemic stroke history, and blood pressure on admission), procedure details, and outcomes. The baseline NIHSS score was used to assess stroke severity. All patients underwent computed tomography (CT) scans immediately and 24 h after MT. The follow-up magnetic resonance (MR) imaging, including the additional sequences of MR angiography and perfusion, was also performed approximately 1 week after MT if the patient cooperated. A non-contrast CT scan was performed immediately to exclude intracranial hemorrhage if any deterioration in the patient's neurological status was observed. Hemorrhagic transformation (HT) was evaluated according to the European Cooperative Acute Stroke Study II (ECASS II) criteria, and symptomatic intracranial hemorrhage (sICH) was defined as a hemorrhage observed on the CT scan accompanied by a deterioration in the patient's neurologic status, defined as an increase in NIHSS score by ≥4 (13). The clinical outcome was assessed using the mRS score; the 90-day mRS score was obtained from the clinic or through telephonic interviews. Successful reperfusion was defined as a modified Thrombolysis in Cerebral Infarction (mTICI) score of 2b/3. FR was defined as a poor clinical outcome (a 90-day mRS of 3–6) despite successful recanalization, and patients were divided into FR and meaningful recanalization groups.

At our institution, multimodal CT-based images are routinely conducted in late time windows in patients with suspected stroke, including non-contrast CT, CTP, and post-processed series. Automated Alberta Stroke Program Early Computed Tomography Scores (ASPECTS) were obtained from a non-contrast CT. Perfusion images were processed using the commercial software RAPID (iSchemaView, Menlo Park, California, USA), which automatically provided colored parametric CTP maps. The ischemic core volume (cerebral blood flow [CBF] < 30%), hypoperfusion volume (Tmax > 6 s), and mismatch volume were obtained from CTP maps. The collateral status evaluated on angiography was divided into two categories according to the American Society of Interventional and Therapeutic Neuroradiology/Society of Interventional Radiology (ASITN/SIR) guidelines: good collaterals (grade 3–4) and poor collaterals (grade 0–2). The imaging parameters were assessed by two experienced neuroradiologists who were blinded to the clinical treatment and outcomes; if any discrepancies arose between the two readers, a consensus was achieved with the help of a third neuroradiologist.

### Endovascular procedure

According to the current guidelines, patients are eligible to receive an intravenous recombinant tissue plasminogen activator before MT within 4.5 h after stroke onset. MT was performed under local anesthesia/conscious sedation. A stent retriever thrombectomy was recommended as the first-line thrombectomy technique, but other devices were also permitted. Rescue therapies were defined as interventions performed after a failed MT, including permanent stent placement, balloon angioplasty, and use of glycoprotein IIb/IIIa antagonists.

### Statistical analysis

Continuous variables are described as means (standard deviation, SD) or medians (interquartile range, IQR) and categorical variables are presented as frequencies (%). The Shapiro-Wilk test and histograms were used to assess the normality of the distributions. A Student's *t*-test or a Mann-Whitney *U*-test was performed to analyze continuous data. The Fisher exact or χ^2^ test was used to analyze categorical data. Significant clinical factors (*P* < 0.1) identified using univariate logistic regression analyses were included in the multivariate logistic regression model to determine odds ratios (ORs) and confidence intervals (CIs). Multicollinearity was assessed by calculating variance inflation factors (VIFs) for variables included in the final model, and substantial multicollinearity was defined as a VIF < 10. Considering that rescue therapies may bring a bias to the results, a sensitivity analysis was undertaken in the subgroup of patients not treated with rescue therapies. Receiver operating characteristic (ROC) curve analyses were applied to identify the effectiveness of significant variables in predicting FR. SPSS 26.0 (IBM, Armonk, NY, USA) and MedCalc (version 11.0, Solvusoft Corporation, Los Angeles, CA, USA) software packages were used for analysis. A *P*-value of <0.05 was considered statistically significant.

## Results

The mean age of the 99 patients who met the inclusion criteria was 72 years (IQR, 66–80) and 50.5% of these patients were women. The median baseline NIHSS score was 16 (11–22), and the median baseline ASPECTS score was seven (IQR, 5–8). The numbers of patients who had occlusions in the ICA (isolated, T- or L-shaped, and tandem occlusions), M1, and M2 were 35 (35.4), 53 (53.5), and 11 (11.1%), respectively. The median volumes of ischemic core and hypoperfusion were 9 mL (IQR, 0–27) and 139 mL (IQR, 89–179), respectively. Intravenous tissue plasminogen activator (tPA) was administered in 20 patients (20.2%) at local hospitals before MT was performed. The etiologies of stroke were cardiac embolism (50, 50.5% of patients), large artery atherosclerosis (33, 33.3% of patients), and undetermined etiology or others (16, 16.2% of patients). The median onset-to-puncture (OTP) and puncture-to-recanalization (PTR) times were 572 min (IQR, 431–793) and 67 min (IQR, 49–100), respectively. Rescue therapy was performed in 24 patients (24.2%), including stent-retriever detachment alone in two, balloon angioplasty alone in 13, and balloon angioplasty plus stenting in 9. The rates of HT and sICH were 40.4% (40/99) and 16.2% (16/99), respectively, and 90-day mortality was observed in 18 patients (18.2%).

FR was observed in 51 patients (51.5%). Table 1 shows a comparison of the characteristics and clinical outcomes between the futile and meaningful recanalization groups. Patients with FR were older (median age of 76 years vs. 68, *P* < 0.001), and a higher proportion of them were women (60.8 vs. 39.6%, *P* = 0.035); those in the FR group also had a higher prevalence of atrial fibrillation (62.7 vs. 33.3%, *P* = 0.003), a lower incidence of smoking history (3.9 vs. 22.9%, *P* = 0.012), a higher median NIHSS score at admission (19 vs. 12, *P* = 0.001), a lower median ASPECTS score (7 vs. 8, *P* = 0.039), and a higher median number of passes per procedure (2 vs. 1, *P* = 0.041). In addition, a larger proportion of patients in the FR group had cardiac embolism stroke (*P* = 0.001) and poor collaterals (*P* = 0.021). Furthermore, there were significant differences in terms of HT, sICH, and mortality rates (*P* < 0.001).

**Table 1:** Comparisons of characteristics and clinical outcomes between futile and meaningful recanalization groups.

**Variables**	**Total (*n* = 99)**	**Futile recanalization** **(*n* = 51)**	**Meaningful** **recanalization (*n* = 48)**	***P-*** **value**
Age (years), median (IQR)	72 (66–80)	76 (71–83)	68 (57–74)	**<**0.001
Female sex, *n* (%)	50 (50.5)	31 (60.8)	19 (39.6)	0.035
Arterial hypertension, *n* (%)	65 (65.7)	36 (70.6)	29 (60.4)	0.287
Diabetes mellitus, *n* (%)	17 (17.2)	11 (21.6)	6 (12.5)	0.232
Myocardial infarctio*n, n* (%)	13 (13.1)	8 (15.7)	5 (10.4)	0.438
Hyperlipidemia, *n* (%)	5 (5.1)	3 (5.9)	2 (4.2)	1.000
Atrial fibrillation, *n* (%)	48 (48.5)	32 (62.7)	16 (33.3)	0.003
History of ischemic stroke, *n* (%)	14 (14.1)	8 (15.7)	6 (12.5)	0.649
Smoking, *n* (%)	13 (13.1)	2 (3.9)	11 (22.9)	0.012
Glucose (mmol/L), median (IQR)	6.4 (5.1–8.4)	6.7 (5.3–8.6)	6.1 (4.6–8.3)	0.786
Baseline SBP (mm Hg), mean (SD)	149 (22.5)	152 (23.2)	144 (21.2)	0.078
Baseline NIHSS score, median (IQR)	16 (11–22)	19 (14–23)	12 (9–17)	0.001
Baseline ASPECTS, median (IQR)	7 (5–8)	7 (6–8)	8 (7–9)	0.039
Ischemic core (mL), median (IQR)	9 (0–27)	9 (0–23)	5 (0–16)	0.056
Hypoperfusion (mL), median (IQR)	139 (89–179)	142 (99–176)	139 (85–186)	0.657
Mismatch (mL), median (IQR)	121 (70–156)	123 (81–146)	126 (68–179)	0.908
Treatment with IV alteplase, *n* (%)	20 (20.2)	9 (17.6)	11 (22.9)	0.191
Local anesthesia, *n* (%)	99 (100.0)	51 (100.0)	48 (100.0)	1.000
Etiology, *n* (%)				0.001
Cardio–embolism	50 (50.5)	34 (66.7)	16 (33.3)	
Large artery atherosclerosis	33 (33.3)	9 (17.6)	24 (50.0)	
Undetermined etiology or others	16 (16.2)	8 (15.7)	8 (16.7)	
Occlusion site, *n* (%)				0.779
ICA	35 (35.4)	17 (33.3)	18 (37.5)	
M1	53 (53.5)	29 (56.9)	24 (50.0)	
M2	11 (11.1)	5 (9.8)	6 (12.5)	
Poor collaterals, *n* (%)	51 (51.5)	32 (62.7)	19 (39.6)	0.021
OTP time (min), median (IQR)	572 (431–793)	604 (445–762)	520 (410–809)	0.949
PTR time (min), median (IQR)	67 (49–100)	65 (52–102)	71 (49–99)	0.798
Number of passes per procedure, median (IQR)	1 (1–2)	2 (1–3)	1 (1–2)	0.041
Rescue therapy, *n* (%)	24 (24.2)	9 (17.6)	15 (31.3)	0.114
HT, *n* (%)	40 (40.4)	30 (58.8)	10 (20.8)	<0.001
sICH, *n* (%)	16 (16.2)	16 (31.4)	0 (0)	<0.001
Mortality, *n* (%)	18 (18.2)	18 (35.3)	0 (0)	<0.001

SBP, systolic blood pressure; DBP, diastolic blood pressure; NIHSS, National Institutes of Health Stroke Scale; ASPECTS, Alberta Stroke Program Early CT Score; ICA, internal carotid artery; OTP, onset to puncture; PTR, puncture to reperfusion, HT, hemorrhagic transformation; sICH, symptomatic intracranial hemorrhage.

After further adjustment of the variables (including age, female sex, smoking, baseline systolic blood pressure, ASPECTS, ischemic core volume, stroke etiology, and poor collaterals) in multivariate logistic regression analyses (Table 2), we found that older age (OR, 1.12; 95% CI, 1.04–1.22; P = 0.005), female sex (OR, 3.79; 95% CI, 1.08–13.40; *P* = 0.038), a higher NIHSS score on admission (OR, 1.11; 95% CI, 1.02–1.22; *P* = 0.023), and an increased number of passes per procedure (OR, 2.07; 95% CI, 1.11–3.86; *P* = 0.023) were independently associated with the occurrence of FR after thrombectomy. Supplementary Table 1 shows multicollinearity testing for the variables included in the final logistic regression model.

**Table 2:** Logistic regression analysis identifying the risk factors associated with futile recanalization.

**Variables**	**Unadjusted OR (95% CI)**	***P*** **-value**	**Adjusted OR (95% CI)**	***P*** **-value**
Age	1.11 (1.06–1.17)	<0.001	1.12 (1.04–1.22)	0.005
Female sex	2.37 (1.06–5.30)	0.036	3.79 (1.08–13.40)	0.038
Arterial hypertension	1.57 (0.68–3.63)	0.288	–	–
Diabetes mellitus	3.33 (0.65–17.15)	0.150	–	–
Myocardial infarction	1.60 (0.49–5.28)	0.441	–	–
Hyperlipidemia	1.44 (0.23–9.00)	0.698	–	–
Atrial fibrillation	3.37 (1.48–7.69)	0.004	–	–
History of ischemic stroke	1.30 (0.42–4.08)	0.650	–	–
Smoking	0.14 (0.03–0.66)	0.013	–	–
Glucose	1.06 (0.78 −1.54)	0.782	–	–
Baseline SBP	1.02 (0.99–1.04)	0.082	–	–
Baseline NIHSS score	1.10 (1.04–1.17)	0.002	1.11 (1.02–1.22)	0.023
Baseline ASPECTS	0.82 (0.68–0.99)	0.043	–	–
Ischemic core volume	1.03 (1.00–1.05)	0.028	–	–
Hypoperfusion volume	1.00 (0.99–1.00)	0.775	–	–
Mismatch volume	1.00 (0.99–1.00)	0.436	–	–
Treatment with IV alteplase	0.72 (0.27–1.93)	0.515	–	–
Etiology (cardio–embolism vs. others)	4.00 (1.73–9.23)	0.001	–	–
Occlusion site of ICA	0.83 (0.37–1.90)	0.665	–	–
Poor collaterals	2.57 (1.14–5.78)	0.022	–	–
OTP time	1.00 (0.99–1.00)	0.747	–	–
PTR time	1.00 (0.99–1.01)	0.806	–	–
Number of passes per procedure	1.49 (1.00–2.24)	0.052	2.07 (1.11–3.86)	0.023

SBP, systolic blood pressure; DBP, diastolic blood pressure; NIHSS, National Institutes of Health Stroke Scale; ASPECTS, Alberta Stroke Program Early CT Score; ICA, internal carotid artery; OTP, onset to puncture; PTR, puncture to reperfusion.

In addition, in the sensitivity analysis that excluded patients not treated with rescue therapies, the results did not change substantially (Supplementary Table 2).

**Figure 2:**
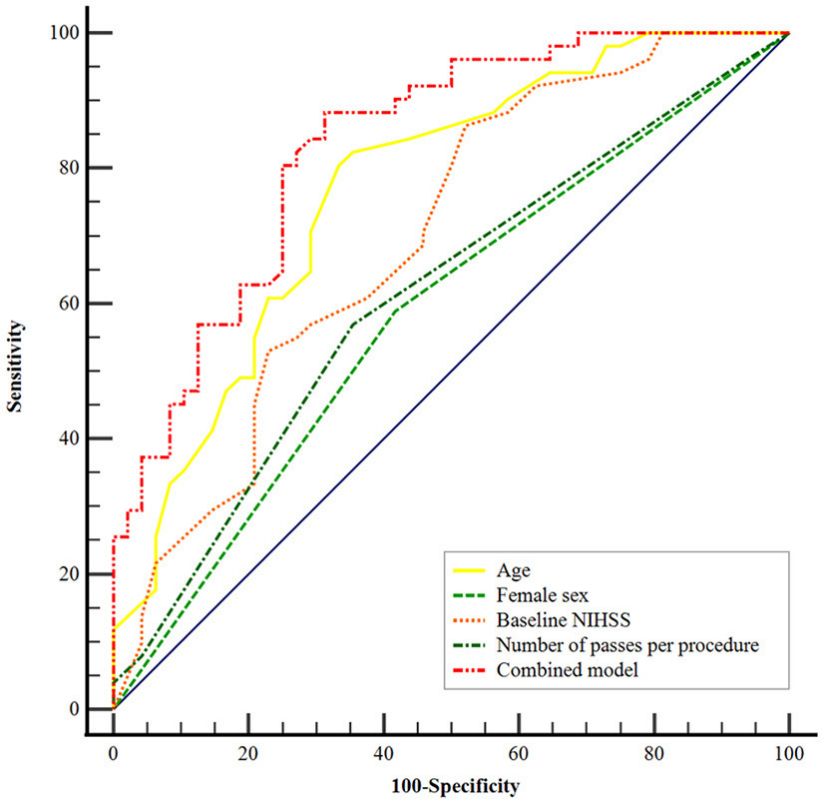
Receiver operating characteristic curves were generated to evaluate the efficacy of age, female sex, baseline NIHSS score, and number of passes per procedure independently and combined for the prediction of futile recanalization after mechanical thrombectomy. The area under the curve of the combined model was 0.84 (95% CI, 0.75–0.90; *P* **<** 0.001). NIHSS, National Institute of Health Stroke Scale.

Using the ROC curves from the logistic regression analysis, we identified the predictive accuracy of age, female sex, NIHSS score on admission, and the number of passes per procedure for predicting FR (Figure 2). The area under the curve (AUC) for age, sex, NIHSS score on admission, and the number of passes per procedure were 0.77 (95% CI, 0.67–0.85), 0.59 (95% CI, 0.48–0.68), 0.70 (95% CI, 0.60–0.79), and 0.61 (95% CI, 0.51–0.71). The model combining age, female sex, baseline NIHSS score, and the number of passes per procedure had the highest AUC (0.84; 95% CI, 0.75–0.90).

## Discussion

In this cohort of patients treated in late time windows, we observed an FR incidence of 51.5% after thrombectomy. The main finding of this study was that advanced age, female sex, a higher NIHSS score on admission, and an increased number of passes per procedure were independently associated with FR after adjusting for confounders. The combination of these independent risk factors increased the model's ability to predict FR in these patients.

The results from the DAWN and the DEFUSE-3 trials led to prolonged time windows for MT in patients with AIS selected by perfusion imaging and changed the guidelines for the early management of these patients (1–3). However, a substantial proportion of patients in both these trials had poor clinical outcomes despite successful recanalization, even after rigorous imaging screening. Reperfusion in delayed time windows may carry harmful consequences, such as severe ischemia-reperfusion injury, and the chances of functional independence may decline. Therefore, predicting FR in patients treated with MT in late time windows may help clinicians select more personalized therapy for patients undergoing MT and identify patients who need other timely adjuvant treatments. Predictors of FR in early time windows have received widespread attention, and several predictors have previously been identified, such as age, female sex, systolic blood pressure upon admission, serum glucose, baseline NIHSS score, time from onset to treatment, and post-procedural complication events (5–12). In this study, we confined our analysis to patients with stroke who underwent treatment in late time windows and identified several clinical markers as independent predictors of poor outcomes after successful recanalization; these predictors could help manage these patients.

Consistent with most previous studies (5, 10–12, 14), we found that advanced age was associated with functional dependence despite successful recanalization, which may be explained by the higher prevalence of underlying diseases, higher incidence of complications, and lower potential for rehabilitation in older age groups compared with younger ones. In addition, older adults are more likely to have leukoaraiosis and poor collateral status than younger individuals, which may be linked to the occurrence of intracranial hemorrhage and poor outcomes after successful reperfusion (15). Nevertheless, older patients could still benefit from endovascular treatment despite their higher rate of FR compared with younger patients. Thus, it seems unjustified to exclude older adults from thrombectomy, but this poor prognostic indicator could help inform discussions with patients' families about the likely prognosis after stroke.

It is generally accepted that the baseline NIHSS score is a strong predictor of FR after thrombectomy. Our study confirmed that a higher NIHSS score on admission (≥12) was associated with FR in delayed windows patients, which was in line with previous studies (5, 7–11, 14). However, Lee et al. found that the clinical benefit of reperfusion after thrombectomy increased with the progression of stroke severity despite the increased rate of FR (8). Similarly, a meta-analysis showed that patients with severe stroke (an NIHSS score of more than 20) experienced greater benefits from thrombectomy than from pharmaceutical treatment (16). This may be because patients with severe stroke tend to have a higher likelihood of functional dependence if untreated, and a proportion of patients with sufficient salvageable brain tissue could recover after reperfusion. According to these findings, when considered as a non-modifiable risk factor, a high NIHSS score should not be considered an exclusion criterion for thrombectomy.

Our analysis also showed that female sex was associated with FR in patients treated in late time windows. The impact of sex on FR is still unclear. Hussein et al. (7) reported similar results: women had a lower rate of favorable outcomes despite successful recanalization compared with men (38.7 vs. 56.5%). In a *post hoc* analysis of the MR CLEAN trial, women had worse treatment outcomes after intra-arterial treatment, although no difference in the recanalization rate was observed (17). In this analysis, the association between female sex and FR was significant after adjusting for confounders. A possible explanation is that women have a higher incidence of atrial fibrillation, and different stroke etiologies may influence the treatment strategies and clinical outcomes (7). This observed difference in treatment outcomes between men and women should be further analyzed with a larger sample size to rule out coincidence.

Several procedure-related factors have been associated with poor outcomes after thrombectomy, including the first-pass effect (18, 19), the number of stent retriever passes (20, 21), and the PTR time (10). The present study showed that an increased number of passes per procedure was associated with 90-day functional dependence despite successful reperfusion after MT. However, the PTR time was not associated with functional dependence in our study. In addition, we did not find a significant association between the OTP time and FR, which was consistent with the data from DEFUSE-3 (22). Despite this, the importance of shortening OTP and PTR time should be stressed in clinical practice.

According to the recently published studies, the effect of collateral status on the functional outcome after recanalization is still controversial. Pan et al. (14) observed a lower rate of FR in patients with good collateral circulation before endovascular treatment. Conversely, a meta-analysis found no significant difference in collateral status between the FR and meaningful recanalization groups (23). Our analysis did not observe a strong association between collateral status and FR after adjusting for the available variables. These contradictory results may be explained by the differences in approaches used to assess collateral circulation and the existence of heterogeneity. In addition, a large final infarct volume was shown to be associated with FR after endovascular therapy in patients with stroke (12). However, no analysis has yet identified an association between preoperative ischemic core volume on CTP imaging and FR. Ribo et al. (24) indicated that patients with lesion core volumes of >39 mL upon admission had poor outcomes after endovascular treatment. By contrast, Heit et al. reported no difference in the ischemic core between the FR and meaningful recanalization groups (22). We found that patients with FR had a slightly larger ischemic core, but this result was not statistically significant. It should be noted that a small ischemic core was observed in most patients included in this study after selection by perfusion imaging, and core volumes larger than 50 mL were rare (10/99, 10.1% of patients). Further studies with a larger sample size are needed to evaluate the prognostic value of these variables (collaterals and core) for futile recanalization.

This study has several potential limitations. First, it has limitations inherent to the retrospective study design. Second, the relatively small sample size may limit the interpretation of the results; some variables with a trend toward significance may show significant associations in analyses with larger sample sizes. Third, post-procedural complications, such as the occurrence of sICH and pneumonia, were not analyzed in the multivariate model because we mainly focused on evaluating preoperative clinical factors and procedural details, and the data on pneumonia were not collected. Finally, the lack of independent core laboratory adjudication for imaging parameters may affect the reliability of the results.

## Conclusions

Although the benefits of MT have been demonstrated in many trials and meta-analyses, the incidence of FR remains a major concern in emerge thrombectomy. In patients with acute stroke treated in late time windows, our results suggest that older age, female sex, higher NIHSS score on admission, and an increased number of passes per procedure are independent predictors of FR after thrombectomy. This finding could help select a population of patients who may potentially benefit more of adjunctive therapies to maximize the benefit of MT, but not for selecting patients for MT treatment. Further studies are needed to validate the predictive value of this model for futile recanalization in this specific subset of acute stroke.

## Data availability statement

The raw data supporting the conclusions of this article will be made available by the authors, without undue reservation.

## Ethics statement

The studies involving human participants were reviewed and approved by the Ethical Standards of the Institutional Research Committee of Jiangsu Province Hospital, the First Affiliated Hospital of Nanjing Medical University (IRB number: 2021-SR-516). Written informed consent for participation was not required for this study in accordance with the National Legislation and the Institutional requirements.

## Author contributions

HN and XL analyzed the data and drafted the manuscript. SL designed the study and helped to revise this manuscript. LZ conceived the study and made final approval of this manuscript. YH, ZJ, YC, and HS helped to perform the analysis with constructive discussions. All authors contributed to the article and approved the submitted version.

## Conflict of interest

The authors declare that the research was conducted in the absence of any commercial or financial relationships that could be construed as a potential conflict of interest.

## Publisher's note

All claims expressed in this article are solely those of the authors and do not necessarily represent those of their affiliated organizations, or those of the publisher, the editors and the reviewers. Any product that may be evaluated in this article, or claim that may be made by its manufacturer, is not guaranteed or endorsed by the publisher.
