# Supplementary material for: Predictors of futile recanalization in patients with acute ischemic stroke undergoing mechanical thrombectomy in late time windows
Source: Front Neurol. 2022 Sep 15;13:958236. doi: 10.3389/fneur.2022.958236 (PMC9519892; doi:10.3389/fneur.2022.958236)
Supplement: Supplementary file 1 [file Data_Sheet_1.PDF]

Table S1 Multicollinearity assessment.

Variables	VIF
Age	1.149
Female	1.155
Atrial fibrillation	2.781
Baseline SBP	1.188
Smoking	1.189
Baseline NIHSS	1.344
Baseline ASPECTS	1.419
Ischemic core volume	1.446
Stroke etiology	2.743
Poor collaterals	1.214
Number of passes per procedure	1.074

VIF, variance inflation factor; SBP, systolic blood pressure; NIHSS, National Institutes of Health Stroke Scale; ASPECTS, Alberta Stroke Program Early CT Score.

Table S2 Risk factors associated with futile recanalization in a sensitivity analysis.

Variables	Excluding patients not treated with rescue therapies (n=75)*
Age	1.11 (1.05-1.19)
Female sex	3.05 (1.12–8.24)
Baseline NIHSS score	1.10 (1.02-1.17)
Number of passes per procedure	1.51 (1.17-1.94)

*Odds ratios (ORs) and confidence intervals (CIs) were calculated by multivariate logistic regression model.
